# *PAX6* analysis of two sporadic patients from southern China with classic aniridia

**Published:** 2012-08-07

**Authors:** Ying Lin, Xialin Liu, Shanshan Yu, Lixia Luo, Xuanwei Liang, Zhonghao Wang, Chuan Chen, Yi Zhu, Shaobi Ye, Hong Yan, Yizhi Liu

**Affiliations:** 1 State Key Laboratory of Ophthalmology, Zhongshan Ophthalmic Center, Sun Yat-sen University, Guangzhou, China; 2 Department of Pharmacology (State-Province Key Laboratories of Biomedicine-Pharmaceutics of China), Harbin Medical University, Harbin, Heilongjiang, China

## Abstract

**Purpose:**

To investigate the paired box 6 (*PAX6*) gene in two sporadic patients from southern China presenting with classic aniridia.

**Methods:**

The two sporadic patients underwent complete physical and ophthalmic examinations. Genomic DNA was extracted from the leukocytes of the peripheral blood collected from the families of the two sporadic patients and 100 unrelated control subjects from the same population. Exons 4–13 of *PAX6* were amplified by polymerase chain reaction (PCR) and sequenced directly. The ophthalmic examinations included best-corrected visual acuity, slit-lamp examination, fundus examination, optical coherence tomography, and Pentacam and Goldmann perimetry.

**Results:**

The two patients were affected with aniridia accompanied by nystagmus. A heterozygous *PAX6* frameshift mutation in exon 7, c.375_376delAG (p.Arg125SerfsX7), was identified in sporadic patient 1 and not in any of the unaffected family members and normal controls. One novel mutation in exon 10, c.868_871dupAGTT (p.Phe291X), was detected in sporadic patient 2. The frameshift mutation we identified has not previously been reported either in China or abroad.

**Conclusions:**

Although *PAX6* mutations and polymorphisms have been reported in various ethnic groups, we report, for the first time, the identification of one new *PAX6* mutation in Chinese aniridia patient.

## Introduction

Congenital aniridia is a rare ocular disease usually caused by mutations in the paired box 6 (*PAX6*) gene, which is located on chromosome 11p13 [1-4]. The disease is characterized by the partial or complete absence of the iris, and is accompanied by other symptoms, including corneal degeneration, cataract, foveal and optic nerve hypoplasia, and nystagmus [5-13]. Two-thirds of aniridia cases are inherited in an autosomal, dominant fashion with variable expressivity, and the other cases are sporadic.

A large number of sporadic cases suggest the diversity of *PAX6* mutations. At present, the *PAX6* mutation database lists more than 500 different mutations of the *PAX6* gene.

This study analyzes the coding sequences of *PAX6* in two sporadic patients with aniridia. One novel *PAX6* mutation was detected in the Chinese population; both mutations were heterozygous.

## Methods

### The aniridia family

Two sporadic patients were diagnosed with aniridia at the Zhongshan Ophthalmic Center.

We performed the ophthalmic examinations as follows:

- Visual acuity was examined using the ETDRS chart (Precision Vision; La Salle, IL).
- An anterior segment photograph was obtained using a BX 900 Slit-Lamp (Haag-Streit; Bern, Switzerland).
- A fundus photograph was obtained using a Heidelberg Retina Angiograph (Heidelberg Engineering; Heidelberg, Germany).
- Optical coherence tomography (OCT) scans (TOPCON; Tokyo, Japan) were used to assess the thickness and pathology of the posterior pole of the retina.
- Anterior segment dimensions were measured with a Pentacam (HR version 70700; Oculus; Weltzar, Germany).

In addition, we performed complete physical examinations of the patients to exclude systemic diseases.

### Sample collection

The two sporadic patients were identified at the Zhongshan Ophthalmic Center. The parents, the brothers and sisters of the two patients, and 100 subjects (25.34±8.63 years old, 45 male) from the same population without diagnostic features of aniridia, were recruited to serve as normal controls. After obtaining informed consent from all participating individuals according to the principles of the Declaration of Helsinki, the peripheral venous blood samples were collected for genomic DNA extraction from the blood leucocytes via standard protocol and procedures.

### Detection of the mutation

All coding exons of the known candidate gene (*PAX6*), as well as their flanking regions, were amplified by PCR with primers [14] (Table 1). The sequencing results were analyzed using Chromas (version 2.3; Technelysium Pty Ltd.; Brisbane, QLD, Australia) and compared with the reference sequences in the database at the National Center for Biotechnology Information (NCBI; NC_000011.9). The superimposed mutant PCR products were sub-cloned into pGEM-T vector (Promega, Madison, WI) and sequenced to identify the mutation.

**Table 1:** Primers used for PCR.

**Exon**	**Forward (5′-3′)**	**Reverse (5′-3′)**	**Product size (bp)**	**Annealing temperature (°C)**
4	TGCAGCTGCCCGAGGATTA	GCACCCCGAGCCCGAAGTC	144	66
5	TCCCTCTTCTTCCTCTTCACT	GGGGTCCATAATTAGCATC	301	58
5 a,6	GCTCTCTACAGTAAGTTCTC	AGGAGAGAGCATTGGGCTTA	457	59
7	AATCCACCCACTGTCCCG	CCAGCCACCTTCATACCG	542	60
8	TCAGGTAACTAACATCGCA	GTTGACTGTACTTGGAAGAA	719	53
9,10,11	GAGGTGGGAACCAGTTTGATG	CAAGCCAATCTCTGTAGTGCG	890	52
12	GCTGTGTGATGTGTTCCTCA	AAGAGAGATCGCCTCTGTG	245	58
13	CATGTCTGTTTCTCAAAGGG	CCATAGTCACTGACTGAATTAACAC	202	58

Summary of the primers and products length used for the amplification of the exons of *PAX6*.

## Results

### Clinical data

The two sporadic patients (case 1 and case 2) studied in this report originated from the southern area of China. Both of them had aniridia and nystagmus without other systemic diseases.

**Figure 1:**
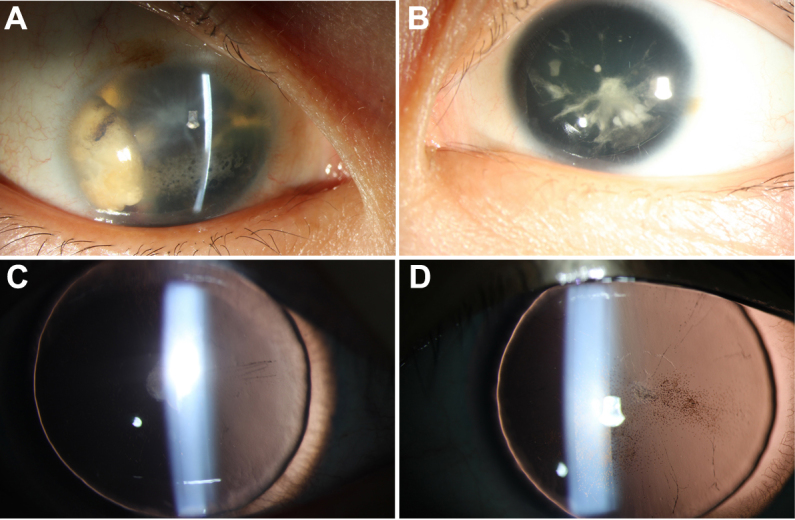
Anterior segment photographs of case 1 and case 2 patients with aniridia are shown. **A**: Shows the case 1 patient (25 years old, male), who had very serious cataracts and corneal degeneration, in addition to aniridia of the right eye. **B**: Shows the left eye of patient 1, who had partial cataracts. **C** and **D**: Show anterior segment photographs of both eyes of case 2. Some pigment particles precipitated in the front of the anterior lens capsule.

**Figure 2:**
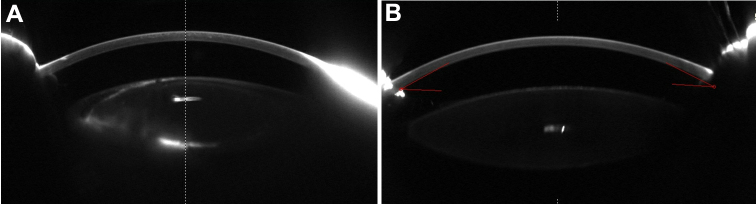
Pentacam photographs of the two patients. **A**: A Pentacam photo shows the anterior segment picture of the left eye of case 1 (with partial cataracts). **B**: A Pentacam photo shows the anterior segment picture of the left eye of case 2.

The right eye of patient 1 (25 years old, male) had very serious cataracts and corneal degeneration (Figure 1A); so, we could not perform all of the examinations; applanation tonometry revealed normal intraocular pressure in both eyes. The widths of the corneas of patient 1 were 10.5 mm (OD) and 10.5 mm (OS), respectively. The axial length of the left eye was 26.02 mm. The lens of the left eye had partial opacities (Figure 1B and Figure 2A). We could not examine the fundus of the right eye due to the cataracts and corneal degeneration. No abnormalities were detected in the retina, choroids, and optic nerve of the left eye. The anterior segment photograph, taken with a Pentacam, is shown in Figure 2A; the anterior chamber depth was 1.93 mm (OS). When this study was performed, the patients could detect only light perception (OD) and hand movement (OS).

The visual acuity of the case 2 patient (8 years old, female), as measured by logarithm of the minimum angle of resolution (Log MAR), was 2.0 (OD) and 2.0 (OS). The widths of the corneas of the case 2 patient were 10.5 mm (OD) and 10.5 mm (OS), respectively. The axial lengths of the eyeballs were 22.45 mm (OD) and 22.59 mm (OS), respectively. Figure 1C,D shows anterior segment photographs of both eyes of the case 2 patient. Some pigment particles precipitated in the front of the anterior lens capsule. No abnormalities were detected in the retina, choroids, and optic nerve. The anterior segment photograph of the left eye, taken with a Pentacam, is shown in Figure 2B. The anterior chamber depths were 2.42 mm (OD) and 2.28 mm (OS), respectively.

### Mutation screening

**Figure 3:**
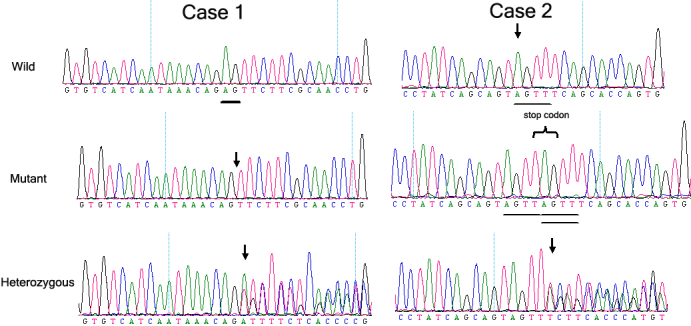
The DNA sequence of a part of the *PAX6* gene in the affected patients and unaffected individuals is shown. Case 1 represents a heterozygous AG deletion at nucleotide 375–376 (c.375_376delAG) in exon 7 of the *PAX6* gene. This frameshift mutation was predicted to cause a PTC seven codons downstream from the ﬁrst new inappropriate codon 125 created by the nucleotide mutation (p.Arg125SerfsX7). The underlining (“Wild” graph) shows the AG in the normal controls, which the affected patients lacked (the arrow pointing to in the “Mutant and Heterozygous” graph). Case 2 represents one novel mutation in exon 10, c.868_871dupAGTT (p.Phe291X); a 4-nucleotide (AGTT) duplication generated a frameshift mutation in exon 10, and this frameshift mutation was predicted to cause a PTC in the codon 291 created by the nucleotide duplication.

In sporadic patient 1, a heterozygous AG deletion at nucleotide 375–376 (c.375_376delAG), in exon 7 of the *PAX6* gene, was confirmed by sequencing (Figure 3). This frameshift mutation is predicted to cause a premature termination codon (PTC) 7 codons downstream from the ﬁrst new inappropriate codon 125 created by the nucleotide mutation (p.Arg125SerfsX7).

One novel mutation in exon 10, c.868_871dupAGTT (p.Phe291X), was detected in sporadic patient 2 (Figure 3); 4-nucleotide (AGTT) duplication generated a frameshift mutation in exon 10, and this frameshift mutation was predicted to cause a PTC in the codon 291 created by the nucleotide duplication. The frameshift mutation we identified has not previously been reported either in China or abroad. No mutations were found in any of the unaffected family members and the normal controls.

## Discussion

In this study, we found two mutations—one in each of the two exons of the *PAX6* gene—that are associated with aniridia: c.375_376delAG and c.868_871dupAGTT. These two mutations, rather than being rare polymorphisms in the normal population, were the causative mutations in the two sporadic patients.

The characteristic of the c.375_376delAG mutation, which was identiﬁed in exon 7 of *PAX6* of the family members, is that it occurred at a hotspot for mutations that had already been shown among other ethnic groups in the PAX6 Allelic Variant Database.

One novel mutation in exon 10, c.868_871dupAGTT (p.Phe291X), generated a frameshift mutation in exon 10, and this frameshift mutation was predicted to cause a PTC in the codon 291 created by the nucleotide duplication. The frameshift mutation we identified has not previously been reported either in China or abroad.

We reviewed the mutations archived in the PAX6 Allelic Variant Database. We found that over three-quarters of aniridia cases are caused by mutations that introduce a PTC into the open reading frame of *PAX6*, just as our study has demonstrated.

To avoid the potentially lethal consequences of producing truncated polypeptides that could interfere with cell functions, mRNAs carrying premature termination codons were rapidly degraded in vivo by a form of RNA surveillance known as nonsense-mediated mRNA decay (NMD) [15,16].

The presence of cataracts was the most common disorder associated with the aniridic patients, and it increased with age [17-20]. So, patient 1 (25 years old) had more serious cataracts than patient 2 (8 years old). There was a relatively high risk of intraoperative and postoperative complications in these aniridic cataract patients. They exhibited fragile anterior lens capsules, as well as either associated lens subluxation or weak zonules. Therefore, preventing the progress of cataracts is of vital importance for patients with aniridia.

In summary, this study identified one novel mutation of *PAX6* in one Chinese sporadic patient with aniridia. This finding expands the mutation spectrum of *PAX6*. In families where aniridia is present, the results of the study are useful and valuable for genetic counseling and prenatal diagnosis that is accompanied by corneal degeneration, eyeball horizontal tremors, and cataracts.
